# Assessing the Mobility of Severe Acute Respiratory Syndrome Coronavirus-2 Spike Protein Glycans by Structural and Computational Methods

**DOI:** 10.3389/fmicb.2022.870938

**Published:** 2022-04-15

**Authors:** Soledad Stagnoli, Francesca Peccati, Sean R. Connell, Ane Martinez-Castillo, Diego Charro, Oscar Millet, Chiara Bruzzone, Asis Palazon, Ana Ardá, Jesús Jiménez-Barbero, June Ereño-Orbea, Nicola G. A. Abrescia, Gonzalo Jiménez-Osés

**Affiliations:** ^1^Structure and Cell Biology of Viruses Lab, Center for Cooperative Research in Biosciences (CIC bioGUNE), Basque Research and Technology Alliance (BRTA), Derio, Spain; ^2^Computational Chemistry Lab, CIC bioGUNE, Basque Research and Technology Alliance (BRTA), Derio, Spain; ^3^IKERBASQUE, Basque Foundation for Science, Bilbao, Spain; ^4^Structural Biology Unit, BioCruces Bizkaia Health Research Institute, Barakaldo, Spain; ^5^Precision Medicine and Metabolism Laboratory, CIC bioGUNE, Basque Research and Technology Alliance (BRTA), Derio, Spain; ^6^Cancer Immunology and Immunotherapy Lab, CIC bioGUNE, Basque Research and Technology Alliance (BRTA), Derio, Spain; ^7^Chemical Glycobiology Laboratory, CIC bioGUNE, Basque Research and Technology Alliance (BRTA), Derio, Spain; ^8^Centro de Investigación Biomédica en Red de Enfermedades Hepáticas y Digestivas (CIBERehd), Instituto de Salud Carlos III, Madrid, Spain

**Keywords:** cryo-EM, molecular dynamics, glycans, SARS-CoV-2, spike

## Abstract

Two years after its emergence, the coronavirus disease-2019 (COVID-19) pandemic caused by severe acute respiratory syndrome coronavirus-2 (SARS-CoV-2) remains difficult to control despite the availability of several vaccines. The extensively glycosylated SARS-CoV-2 spike (S) protein, which mediates host cell entry by binding to the angiotensin converting enzyme 2 (ACE2) through its receptor binding domain (RBD), is the major target of neutralizing antibodies. Like to many other viral fusion proteins, the SARS-CoV-2 spike protein utilizes a glycan shield to thwart the host immune response. To grasp the influence of chemical signatures on carbohydrate mobility and reconcile the cryo-EM density of specific glycans we combined our cryo-EM map of the S ectodomain to 4.1 Å resolution, reconstructed from a limited number of particles, and all-atom molecular dynamics simulations. Chemical modifications modeled on representative glycans (defucosylation, sialylation and addition of terminal LacNAc units) show no significant influence on either protein shielding or glycan flexibility. By estimating at selected sites the local correlation between the full density map and atomic model-based maps derived from molecular dynamics simulations, we provide insight into the geometries of the α-Man-(1→3)-[α-Man-(1→6)-]-β-Man-(1→4)-β-GlcNAc(1→4)-β-GlcNAc core common to all *N*-glycosylation sites.

## Introduction

Since the emerging of severe acute respiratory syndrome coronavirus-2 (SARS-CoV-2) more than 900 cryo-EM related entries have been deposited in the Electron Microscopy Database (EMDB); about 600 targeted spike protein alone or in complex with ligands and/or antibodies. It has been shown structurally that the interaction with the cellular receptor ACE2 is mainly protein-protein and not strictly mediated by glycans. However, glycosylation of the SARS-CoV-2 spike is emerging as playing an important role in early attachment across different cell types (Wang et al., 2021). The interaction of the receptor binding domain (RBD) glycans with different human lectins expressed in different organs and tissues that may be affected during the infection process, has been shown, and recent studies demonstrated how lectins enhance SARS-CoV-2 infection (Lenza et al., 2020; Lempp et al., 2021). Also, density and type of glycosylation (so called the “glycan shield”) critically impact on the antibody neutralization mechanism and on the development of suitable vaccines (Casalino et al., 2020). The difficulty to over-express the S protein and the RBD in different cell-types has biased all current structural research to three major mammalian cell lines HEK293, CHO, and Vero and one insect BL9 type (Allen et al., 2021). Efforts have been made to show that current recombinant and vaccine-produced S protein mimic the native viral glycoprotein, but further research is needed (Allen et al., 2021; Wang et al., 2021; Watanabe et al., 2021). Other glycomics studies of the spike protein purified from sputum of infected patients have shown that the glycosylation pattern is similar to the recombinantly produced S protein (Tian et al., 2021). The possibility of alternative glycan sequences for the same site challenges the understanding of the influence of glycosylation in physio-pathological processes. Furthermore, the fact that different tissues and cell types possess their own glycosylation signatures generates a combinatorial complexity to the glycans display that it is challenging to untangle. The advances in cryo-EM have indeed enormously impacted on our structural and dynamic understanding of the viral spike (Stuart et al., 2016; Subramaniam, 2020; Rapp et al., 2022). Recent studies based on the chemical structures of the most populated *N*-glycans derived from glycoanalytic data, have combined computational simulations with existing cryo-EM maps in order (*i*) to develop a fully glycosylated SARS-CoV-2 spike protein model; (*ii*) to map epitopes not shielded by the highly flexible glycans, (*iii*) to reveal their role in modulating the RBD dynamics (Casalino et al., 2020; Woo et al., 2020; Sikora et al., 2021) and (*iv*) to analyze their contribution to interaction with host cell receptors (Kapoor et al., 2021). Although the detailed determination of the glycan structure by cryo-EM (and X-ray crystallography) remains challenging due to the glycans’ mobility (Atanasova et al., 2020), when visible the corresponding densities are the result of the averaging of a “predominant” glycan conformation. The local mechanical flexibility of a carbohydrate polymer is dictated by its primary and secondary structures at the single-linkage level (Anggara et al., 2021) a fact that amplifies the relevance of tissue-specific glycosylation and the structural location of the attachment site in modulating the infection process (not only in SARS-CoV-2).

In this study we sought to use our cryo-EM map at 4.1 Å resolution, reconstructed from a limited number of particles, as a means to explore the implications of oligosaccharide modifications on glycan flexibility as derived from molecular dynamics (MD) simulations. Apart from showing that our low resolution cryo-EM map is informative on the glycans presence and dynamics, we found that in MD trajectories the glycans modeled using the prevalent structure observed in previous studies for defined sites in the S2 domain of the S protein (Casalino et al., 2020) are more congruent with the cryo-EM map than when the same glycans are modified by defucosylation, sialylation and addition of terminal LacNAc units. This finding suggests that the original glycosylation pattern might be dominant in our recombinantly expressed and structurally characterized spike protein.

## Methodology

### The Ectodomain of the S Protein Production and Purification

The ectodomain of the S protein (ecto-S; BEI construct NR-52394) was expressed by transient transfection of HEK293F suspension cells and purified from clarified cell supernatants 7 days post-transfection using a nickel affinity column and size-exclusion chromatography as previously described (Stadlbauer et al., 2020).

### The Ectodomain of the S Protein Sample Preparation and Cryo-EM Data Collection

0.06 mg/mL of ecto-S in saline buffer (20 mM Tris-HCl, pH 8.0 and 300 mM NaCl) was deposited onto a 300 mesh, R 1.2/1.3 Quantifoil grid with continuous carbon support (Electron Microscopy Sciences) that had been previously glow-discharged (37 s at 8 mA). The samples were vitrified using a Vitrobot (Mark III), after blotting for 3 s at 14°C and 100% humidity. Movies were collected on a Titan Krios operating at 300 kV using the EPU automated data collection software and recorded on a Gatan K3 Summit direct electron detector operating in counting mode. Images were recorded at a nominal magnification of 105,000× (super-resolution 0.41 Å/pixel) with a defocus range of −0.8 to −2.3 μm. Two dataset were collected; the first dataset with a dose of 1.24 e-/Å^2^/frame which resulted in 49.6 e-/Å^2^ total dose over 40 frames while the second one with a dose of 1.04 e-/Å^2^/frame which resulted in 52 e-/Å^2^ total dose over 50 frames (Supplementary Table 1).

### Image Processing, 3D Reconstruction, and Model Refinement

Frames were motion corrected using MotionCorr2.1 and the defocus of the resulting individual micrographs was estimated using CTFFIND4 (Rohou and Grigorieff, 2015; Zheng et al., 2017). Initially, the processing of the two datasets was carried out independently. Particles were picked in a reference-free manner using Topaz and crYOLO software (Wagner et al., 2019; Bepler et al., 2020). Particles were extracted, binned ×4 (1.66 Å/pixel), and subjected to iterative rounds of reference-free 2D and then 3D classification to identify differential class averages in RELION-3 (Zivanov et al., 2018) (as reference map we used EMD-21452 filtered to 60 Å resolution). The resulting analysis of the individual datasets led to their merging (65,372 particles). 3D classifications were performed with C1 and C3 symmetries and followed up with 3D refinement routines as we could detect both states: (*i*) one RBD up and (*ii*) three RBD down. We continued the processing of the ecto-S with the three RBD in down conformation in cryoSPARC in C3 symmetry importing 27,668 particles from a previous RELION run (Punjani et al., 2017). An *ab-initio* model was generated in cryoSPARC and a 3D classification with two classes resulted in one “junk” class (4,814 particles) and one “good” class (22,854 particles). Particles in the “good” class were refined first using the homogenous refinement protocol (4.39 Å; Bfactor −150.5 Å^2^) and subsequently using a non-uniform refinement (Punjani et al., 2020) (4.10 Å; Bfactor −157.5 Å^2^; 4.06 Å with auto tightening; default settings) (Supplementary Figure 1). The model PDB ID 6XR8 was chosen for the fitting as it presented a more complete description of the experimental sugar *versus* the corresponding density (Cai et al., 2020). This was initially fitted as a rigid-body into our cryo-EM density and minimized with NCS and secondary structure restraints (three cycles) leading to an overall CC = 70% (Supplementary Table 1), then the downstream analysis focused on the glycan density and their structures.

### Comparison Across the Ectodomain of the S Protein Cryo-EM Maps

**FIGURE 1:**
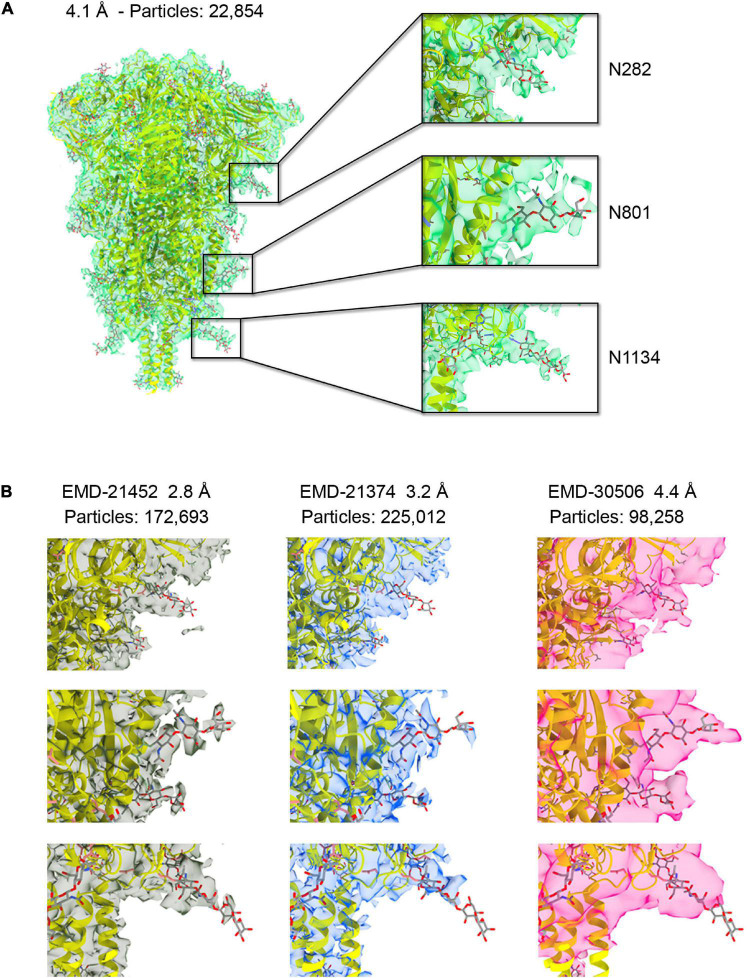
**(A)** The 4.1 Å resolution cryo-EM map of severe acute respiratory syndrome coronavirus-2 (SARS-CoV-2) spike ectodomain shown as transparent green isosurface depicted with a sdLevel = 3 in Chimera X (Pettersen et al., 2021) fitted with the refined structure of the closed, prefusion trimer (PDB ID 6XR8), with protein in cartoon and glycan in sticks. Insets show selected *N*-glycosylation sites along the trimeric spike with different densities features displayed at a sdLevel = 3 (not to scale). **(B)** Comparison of the density features for the same sugars shown in the insets **(A)** for available maps at higher resolution and at similar resolution to ours (not to scale).

To inspect the interpretability of the density corresponding to the glycans in our map, different B-factors were applied: −78.5 and −100 Å^2^. Then, to compare our cryo-EM density with the available higher resolution maps, the power spectra of these maps were adjusted to our map using RELION-3 (Figure 1). In the case of the ecto-S map whose protein was expressed in insect cells this was directly compared since it reached 4.4 Å resolution.

### Model Building for Molecular Dynamics Simulations

Four fully glycosylated spike protein models in the closed conformation were built from PDB structure 6XR8 (trimer with all receptor binding domains in RBD-down conformation) (Cai et al., 2020). This reference structure presents four missing loops, corresponding to residues H69-K77, L244-S254, T618-W633, and T676-S689. These missing loops were taken from a fully glycosylated structure available from the CHARMM-GUI archive (6VSB model 1_1_1) (Jo et al., 2008; Woo et al., 2020) and grafted onto the reference structure to obtain complete chains from residue Q14 to P1162. These models are based on the cryo-EM structure identified by the PDB code 6VSB. This cryo-EM structure has played a fundamental role in the development of computational approaches to understand dynamic and structural implications of glycosylation, most notably by Amaro et al. (Casalino et al., 2020; Sztain et al., 2021). The initial 13 residues at the flexible N-terminal were not modeled. Glycans were built and added to the 57 glycosylation positions (19 for each chain of the trimer) using the GLYCAM-web glycoprotein builder (Woods, 2005). Missing hydrogens, protonation states of titratable residues and histidine tautomers were added and assigned with the *tleap* tool in AMBER (Götz et al., 2014) at neutral pH. This model was named M0 and constituted our glycan reference structure.

**FIGURE 2:**
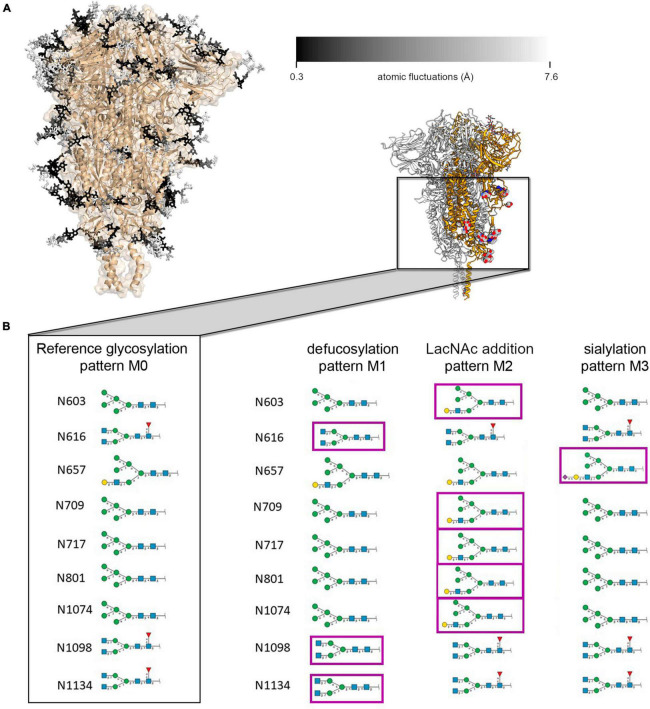
**(A)** Representation of ecto-*S* glycan mobility in model M0 along a 100 ns MD simulation. Glycans (in sticks) are color-coded according to the average mobility (RMSF) of each individual carbohydrate, dark to light corresponding to rigid (0.3 Å) to flexible (>7.6 Å). **(B)** Schematic representation of the glycan structures at each site on the spike protein S2 domain for the model M0 and the modifications introduced in M1, M2, and M3 models; the inset shows the localization on the spike protein of the modified glycans (in balls and sticks).

Then, modifications were introduced: defucosylation at positions N616, N1098, N1134, sialylation at position N657, and addition of terminal LacNAc to high-mannose glycans at positions N603, N709, N717, N801, and N1074 to generate models M1, M2, and M3, respectively (Supplementary Figure 2). These positions are located in the S1 (N603, N616, and N657) and S2 (N709, N717, N801, N1074, N1098, and N1134) subunits of the spike protein trimer (Figure 2A). The selection of these sugar sites was based on the available information on the conformational flexibility of the spike protein and glycosylation pattern density. The head region of the S protein (S1) undergoes conformational transitions at the RBD domains, that can assume two different conformations (“up” and “down”), and shows a higher glycosylation density than the S2 domain (Casalino et al., 2020; Wrapp et al., 2020). It was reasoned that focusing the comparison with the cryo-EM maps on the final region of the S1 domain and the S2 domain, would allow analyzing flexibility effects originating purely from the glycans, and not from conformational transitions of the underlying protein, such as the “up” to “down” switch of the RBD splendidly described by Amaro et al. (Sztain et al., 2021). Also, it was reasoned that focusing on a less densely glycosylated region would allow analyzing intrinsic glycan flexibility with less interference from glycan-glycan contacts.

### Molecular Dynamics Simulations

Molecular dynamics simulations were carried out with AMBER 20 suite (Götz et al., 2014) using the ff14SB force field for the protein (Maier et al., 2015) and GLYCAM 06j-1 for glycans (Kirschner et al., 2008). Glycosylated spike protein models were immersed in a water box with an 8 Å buffer distance from the solute of TIP3P water molecules (Jorgensen et al., 1983) and neutralized by adding explicit Na^+^ counterions. The choice of the size of the buffer distance was dictated by the need of computational efficiency, as the solvated system amounts to more than 630,000 atoms. The total charge of the models before neutralization is –6 for variants M0, M1, and M2, and −9 for M3 due to the presence of sialylated glycans in the latter. A two-stage geometry optimization approach was performed. The first stage minimizes only the positions of solvent molecules and ions, and the second stage is an unrestrained minimization of all the atoms in the simulation cell. The systems were then heated by incrementing the temperature from 0 to 300 K under a constant pressure of 1 atm and periodic boundary conditions. Harmonic restraints of 10 kcal mol^–1^ Å^–2^ were applied to the solute, and the Andersen temperature coupling scheme (Andersen, 1980) was used to control and equalize the temperature. The time step was kept at 1 fs during the heating stages, allowing potential inhomogeneities to self-adjust. The SHAKE algorithm was employed for further equilibration and production with a 2 fs time step (Miyamoto and Kollman, 1992). Long-range electrostatic effects were modeled using the particle mesh Ewald method (Darden et al., 1993). A cutoff of 8 Å was applied to Lennard-Jones interactions. Each system was equilibrated for 2 ns at constant volume and temperature of 300 K. To prevent substantial structural deviation from the reference structure, harmonic restraints of 10 kcal mol^–1^ Å^–2^ were imposed on the protein for the whole simulation, except for the four flexible loops that are not resolved in the reference cryo-EM structure, corresponding to residues H69-K77, L244-S254, T618-W633, and T676-S689. Production simulations were run as a single 100 ns trajectories for each model.

To verify that the spike protein models do not exceed the simulation box owing to glycan flexibility, their size along the three Cartesian coordinates was estimated for selected frames of the MD simulation of the four models (M0–M3) and compared it to the size of the simulation box (which is fixed in the production in the NVT ensemble). The restraints applied to protein atoms guarantee that the orientation of the model is constant along the trajectory. To estimate the size of each glycoprotein, the distances between all pairs of C1 carbons of terminal glycans were computed along each coordinate for 20 evenly sampled frames (every 5 ns) from the production simulation and compared the maximum value with the corresponding box side (Supplementary Tables 2–5). In all cases, the estimated size of the system is lower than the box size by at least 15 Å, which ensures that the model stays within the simulation box along the whole trajectory.

To assess how the conformation of the glycosidic linkages at the glycans common core are affected by the interplay of glycan-glycan and protein-glycan interactions, MD simulations of the free glycan core in solution were performed. Thus, a model of the common α-Man-(1→3)-[α-Man-(1→6)-]β-Man-(1→4)-β-GlcNAc(1→4)-β-GlcNAc core linked to a single asparagine residue capped with N-terminal COMe and C-terminal NHMe groups was built. The same simulation parameters employed for the MD simulations of the spike protein trimers were used, with the exception of the size of the solvent box (a 10 Å buffer of TIP3P water molecules was used), the lack of positional restraints in the production run, and simulation time (500 ns). MD breaks the three-fold symmetry of the spike protein, as every chain moves independently of each other. The fact that MD does not introduce dramatic distortions of the trimer structure in the equilibration step, at which the system undergoes significant volume variations, was ensured by computing the RMSD of the models before and after the equilibration (Supplementary Table 6).

### Analysis of Glycan Molecular Dynamics Trajectories Across the M0, M1, M2, and M3 Glycan Models

The flexibility of each carbohydrate unit along the MD simulations was evaluated by computing the atomic positional fluctuation (also known as root-mean-square fluctuations, RMSF) using the *cpptraj* tool in AMBER (Roe and Cheatham, 2013):

$A\,t\,o\,m\,F\,l\,u\,c\,t_{i}\,\text{or}\,R\,M\,S\,F_{i}=\sqrt{\left\langle {\left(x_{i}-\left\langle x_{i}\right\rangle \right)}^{2}\right\rangle }$

where *x*_*i*_ are the atomic positions and averaging is over the considered frames. Atomic positional fluctuations were combined in per-residue mass-weighted averages:


$$\left\langle F\,l\,u\,c\,t\right\rangle =\frac{\sum_{i}A\,t\,o\,m\,F\,l\,u\,c\,t+M\,a\,s\,s_{i}}{\sum_{i}M\,a\,s\,s_{i}}$$

This flexibility analysis was performed on the whole trajectory: 5,000 frames sampled with an even stride (every 0.02 ns) from the 100 ns production simulation were considered and aligned to the first one prior to RMSF calculation. The solvent accessible surface area (SASA) was evaluated with the *surf* module of *cpptraj* on a subset of 100 frames sampled along the whole trajectory with an even stride (every 1 ns) using a probe radius of 1.4 Å. Glycan shielding percentage was computed as follows: for each MD simulation frame, the SASA value of its protein residues was subtracted from the SASA of a reference spike protein structure with the same aminoacidic composition but not glycosylated. In this way, an instantaneous glycan shielding percentage is computed for each frame, instead of accumulating glycan coverage from multiple frames.

For the cross-correlation analyses (*vide infra*) another subset of 1,000 frames sampled along the whole trajectory with an even stride (every 0.1 ns) was generated. Due to the three-fold symmetry of the cryo-EM structure, the coordinates for each protein monomer were further extracted from the 1,000 MD frames to generate a total set of 3,000 geometries per atomic model.

### Glycan Dynamics in the Context of the Ectodomain of the S Protein Cryo-EM Density

The individual frames composing the trajectories derived from the MD simulation of the individual M0, M1, M2, and M3 models were fitted into our density map using Chimera X (Pettersen et al., 2021). The Electron Microscopy Data Analytical Toolkit (EMDA) was used for estimating the local correlation between the full map and the atomic model-based map at positions corresponding to the atoms in the glycan sugars (Warshamanage et al., 2022). Cross-correlation values were averaged over all non-hydrogen atoms in the individual glycans or over the entire glycan chain.

## Results and Discussion

### Cryo-EM Map of the Ectodomain of the S Protein at 4.1 Å Resolution

The cryo-EM density of the ectodomain of SARS-CoV-2 spike protein was reconstructed from 22,854 particles and reached 4.1 Å resolution (Figure 1A and Supplementary Figure 1). This number of particles represents about 10% of those contributing to EMD-21374 at 3.2 Å, about 13% of those contributing to EMD-21452 at 2.80 Å and about 2.5% of those contributing to EMD-22251 at 2.4 Å resolution (Walls et al., 2020; Wrapp et al., 2020; Zhou et al., 2020). Until recently and to the best of our knowledge – monitoring the Electron Microscopy Data Bank (EMDB) entries only for the S protein structure in closed conformation – our map was the one reconstructed with lowest number of contributing particles. And yet, despite the limited resolution, the map is informative and descriptive of the major structural features noted in higher resolution structures. The fitting and refinement of the atomic model PDB ID 6XR8 (Cai et al., 2020) into the electron density led to a good map-model agreement (CC = 70%) (Figure 1A and Supplementary Table 1). Notably, the density for some of the nineteen glycans in the construct was readily visible in particular for glycans located in the S2 region (Supplementary Figure 3). While the interpretability of critical regions of cryo-EM maps at high-resolution recapitulates the global and local application of B-factor/sharpening as shown in previous test cases also with SARS-CoV-2 structures (Kaur et al., 2021; Sanchez-Garcia et al., 2021), we show that the assessment of the glycan density and their mobility on the spike surface can be derived from a medium-resolution map obtained from a streamlined number of contributing particles. This might serve as further evidence for the implementation of a cryo-EM pipeline for the evaluation of the glycan shielding of targeted protein constructs.

### Comparison of the Densities Corresponding to Glycans Across Cryo-EM Maps of the Ectodomain of the S Protein

The glycan shield in viral proteins greatly influences the recognition of epitopes by the immune system and in some cases when the shield itself is highly dense, as in the case of glycoprotein gp120 on the surface of HIV-1, carbohydrates can be targeted for antibody recognition (Trkola et al., 1996; Kunert et al., 1998). Therefore, rapid structural assessment of the glycan shield can foretell the challenges ahead for an antibody-based therapeutic strategy. To compare the interpretability of the density corresponding to the glycans across our cryo-EM map and the deposited original high-resolution maps EMD-21452 (2.8 Å), –21374 (3.2 Å) we first adjusted the power spectra amplitudes of the latter two to our map (Scheres, 2012). The spike protein samples used for the above cryo-EM reconstructions were produced in the mammalian cell system HEK293. We also compared our map with a fourth one, EMDB-30506 at 4.4 Å resolution (98,258 contributing particles) whose sample was produced in *Trichoplusia ni* insect cells. Differently than mammalian cells, insect cells have a limited capacity to produce glycans with terminal sialic acid (Marchal et al., 2001). Sialyation is most abundantly found at sites N17, N74, N165, N331, N1098, and N1194 within the spike (Watanabe et al., 2020). In our medium-resolution map glycans linked to N709, N717, N801, N1074, N1098, and N1134 were those that showed the most order in their corresponding cryo-EM density, in particular they show good clarity for two GlcNAc and a mannose before bifurcation (Figure 1A and Supplementary Figure 3). We noted, however, that density of the corresponding glycans across the four maps and in correspondence of the S1 and S2 regions were all consistent and with a gradient of increasing mobility from the connector domain (CD) toward the N-terminal domain (NTD) and receptor binding domain RBD (Figure 1B). This obviously reflects a convolution effect of protein dynamics with the intrinsic carbohydrate flexibility, but it also prompts that independently derived spike cryo-EM maps snapshot glycan conformations that are statistically more favorable in time and space than others.

### Influence of Glycan Identity on Mobility

X-ray and cryo-EM derived structures (each in its own way) provide average static snapshots of the targeted macromolecule. In this averaging process either *in cristallo* or through 3D reconstruction methods, flexible parts (carbohydrates even more) become blurred. On the other hand, MD provides a dynamic picture of the glycan shield around the ecto-S with important implications for antibody neutralization (Casalino et al., 2020; Sikora et al., 2021). We first analyzed the flexibility of each carbohydrate constituting the glycan shield at each glycosylation position as described by Amaro and co-workers (Casalino et al., 2020). In order to maintain consistency with the three-fold symmetry (C3) of the cryo-EM map and facilitate further analysis, we modeled the same glycan at each given position of the three protomers (Figure 2A). Besides this original model (M0), we introduced plausible modifications on selected glycans which could be compatible with available mass spectrometry data reporting on the heterogeneity of spike protein glycosylation (Figure 2B; Watanabe et al., 2020). Hence, in variant M1 the glycans linked to residues N616, N1098, and N1134 were defucosylated. In variant M2, *N*-acetyllactosamine (LacNAc) units were added at the end of the α3-branch of the high mannose glycan at positions N603, N709, N717, N801, and N1074. Finally, the α3-branch at position N657 was sialylated (variant M3). In all models, the glycosylation pattern was identical for the three protomers. Overall, the flexibility of the glycans at each glycosylation position was interrogated 12 times through MD simulations (three glycoprotein chains for each of the four M variants). In this way, we obtain a cumulated sampling time of 1.2 μs for each glycosylation position, thus covering a wide variety of glycan-glycan and protein-glycan interactions and terminal glycan modifications. This strategy of analyzing frames accumulated from different glycan identities and trimer chains permits reducing the impact of the intrinsically low conformational sampling achieved by our short individual simulations, by exploring a variety of local environments, and averaging glycan conformations from multiple interaction contexts allowing for different degrees of mobility (Supplementary Figures 4–7). We calculated a similar dynamic glycan shielding of around 13% for the four spike protein variants at each simulation frame (Supplementary Table 7, see “Methodology” section for details on the calculation of glycan shielding). A general observation from our simulations was that, irrespective of the nature of the glycans or the chain they are located, local glycan-glycan and glycan-protein interactions affect flexibility along the whole glycan chain. Given their length, branching and intrinsic flexibility, glycans tend to randomly form local clusters and adhere to the protein surface, thus affecting their local mobility from one simulation to another. This result reveals the need for an efficient sampling strategy when addressing glycan flexibility in large, complex and densely glycosylated proteins, which still poses a significant computational challenge.

**FIGURE 3:**
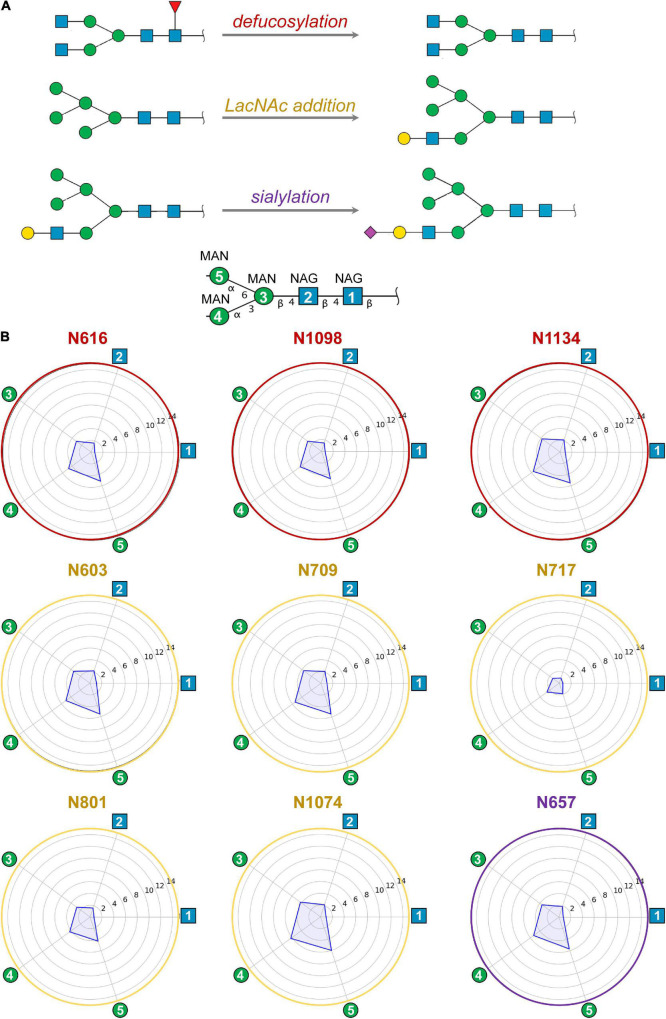
**(A)** Modifications of selected ecto-S glycans with respect to model M0 analyzed by MD simulations: defucosylation (positions N616, N1098, and N1134; model M1), addition of terminal LacNAc (positions N603, N709, N717, N801, and N1074; model M2) and sialylation (position N657; model M3). **(B)** Computed mobility of glycans from MD simulations. For each glycosylation position, the mobility of core carbohydrates is computed as the atomic positional fluctuation (RMSF) in Å. The first two *N*-acetylglucosamines (NAG) and three mannoses (MAN) – i.e., the glycan core – have been considered as they are shared among all glycans. Atomic fluctuations are presented as radial plots. The mobility of the five carbohydrate units is represented anticlockwise starting from the first NAG.

No clear trend was observed regarding the effect of glycan terminal modifications on the flexibility of the glycosylation core (i.e., the five common carbohydrates through which the glycan is attached to the protein, see Figure 3 and Supplementary Figures 4–7), suggesting that for a densely coated glycoprotein, the chemical identity of the glycans terminal region affects glycan core flexibility only indirectly by establishing multiple transient interactions. Figure 3 shows the cumulated mobility of each individual core carbohydrate at each glycosylation position computed from the MD simulations of M0, M1, M2, and M3. Comparison across different glycosylation positions reveals large differences in mobility depending on the morphology of the underlying protein and the glycan density of the surroundings. We detected that positions N234 and N717 are especially rigid. Of note, position N234 has been attributed a structural role (Casalino et al., 2020). Similarly, the large flexibility at N74 reflects the fact that this glycosylation position is located on a flexible loop.

### Reconstruction of Plausible Glycan Conformations From Cryo-EM and MD Simulations

**FIGURE 4:**
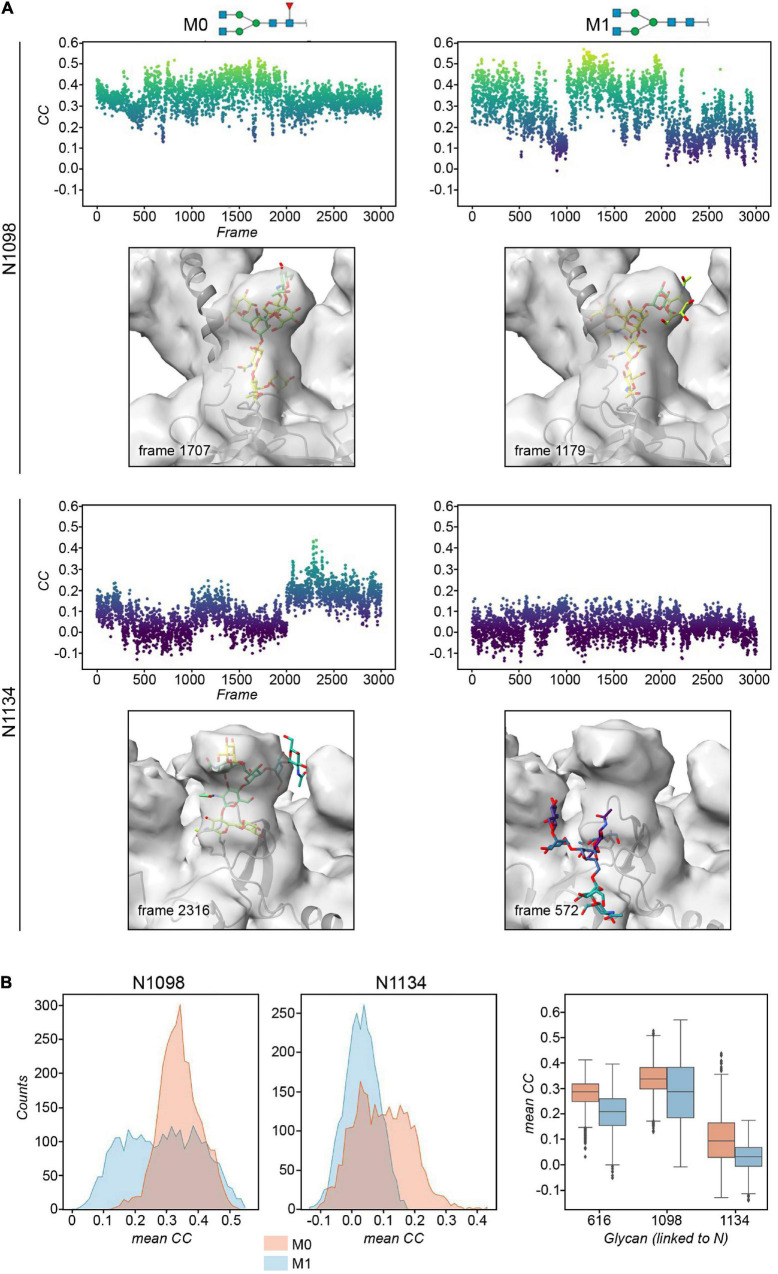
**(A)** Plots of the real-space correlation (CC) of the glycan at site N1098 (*top*) and N1134 (*bottom*) across all the 3,000 frames (each monomer is considered independent) for models M0 (*left*) and M1 (*right*, defucosylated). Below the plot, the glycan structure with the highest real-space cross correlation value in the trajectory is shown within the cryo-EM map. **(B)** Histograms and box plots of the above cross correlation values for the glycans linked to N1098 and N1134 for the M0 and M1 constructs. See also Supplementary Figures 8, 9.

**FIGURE 5:**
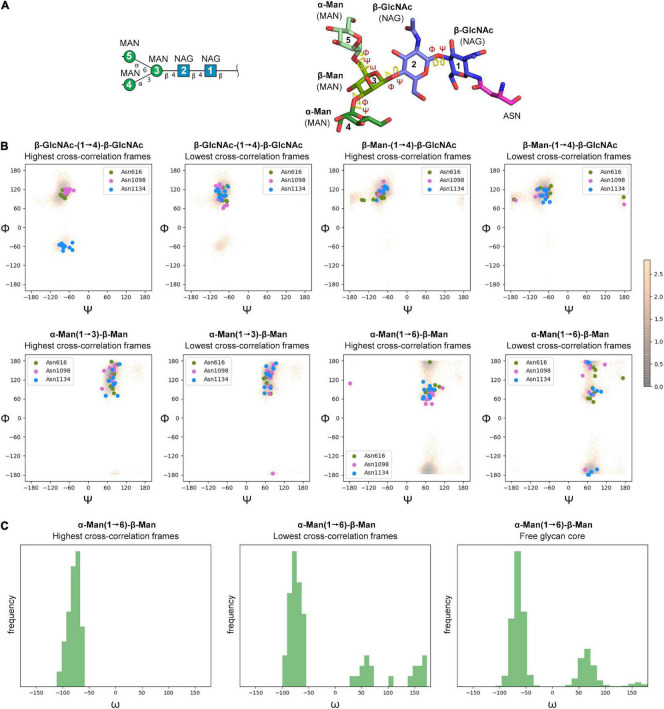
**(A)** Symbol Nomenclature for Glycans (SNFG) and three-dimensional representations of the glycan core analyzed by MD simulations. **(B)** Φ/Ψ plots for the glycosidic bonds in the glycan core of selected glycosylation positions (N616, N1098, and N1134) corresponding to MD snapshots that scored the highest (*n* = 10) and the lowest (*n* = 10) real-space correlation (CC) with cryo-EM density (colored circles), and the free glycan core in solution (density map color-coded according to conformational energy in kcal mol^– 1^ derived from population analysis though a Boltzmann distribution at 25°C). **(C)** Histograms for the ω dihedral angle in the branching mannose of the glycan core of selected glycosylation positions (N616, N1098, and N1134) corresponding to MD snapshots that scored the highest (*n* = 10) and the lowest (*n* = 10) real-space correlation (CC) with cryo-EM density, and the free glycan core in solution.

The cryo-EM maps of the ecto-S unequivocally show that the density corresponding to the glycans of the S2 domain (N709, N717, N801, N1074, N1098, and N1134) is more visible than the others – most of their tubular shaped density projects outward radially from the protein backbone (Figure 1A). This density has been modeled with two GlcNAc and a mannose moiety in the different deposited atomic models and it was indeed inferred that these glycan subunits are quite rigid. Fitting in density of additional monosaccharides is challenging as the density becomes weaker as one moves away and yet in the 4.1 Å resolution map the density further protrudes for defined sites (Supplementary Figure 3). So, opposite to the real-space refinement procedure which optimizes the fitting of a “model” of a protein/glycan to an electron density map, we sought to filter the glycan conformations explored by the MD simulations using the constraints offered by the experimental cryo-EM map to grasp possible glycan chemical signatures that would favor the adopted conformation in the map. This filtering was performed by extracting the individual snapshots from the M0, M1, M2, and M3 trajectories and estimating the real-space correlation between each carbohydrate conformation at a site of interest and the corresponding cryo-EM density by means of the EMDA software (see Methods; Warshamanage et al., 2022). We analyzed all nine glycosylation positions, grouping them according to the type of chemical modification. The real-space correlation at sites N1098 and N1134 (those sugars for which a stronger density is visible) is shown in Figure 4, while the corresponding analysis for the rest of the glycosylation positions is shown in Supplementary Figures 8, 9. For N1098 and N1134, of all the conformations explored only a small selection would fulfill the map. While general rules cannot be drawn it is clear that the presence of a fucose alters the mobility of the glycan. In the case of site N1098 the fucose provides a narrow distribution of poses around the highest CC (Figure 4B, left) while in the case of site N1134 it practically leads to a broad distribution of glycan conformations that, however, on average stays in density more than in the case of the defucosylated variant (Figure 4B, center and right). In the case of M2, the model-map correlation is also lower than for the original glycans in the M0 model while for the M3, the sialylation practically does not alter the average presence of the glycan in the map (Supplementary Figures 8, 9). We cannot exclude possible protein and glycan neighboring effects modulating the adopted conformation along the trajectories. In the case of M0 we also investigated those snapshots that scored the highest (*n* = 10) and the lowest (*n* = 10) real-space correlation and analyzed their glycosidic linkage torsions to assess whether low correlation can arise from violations of the canonical, low energy conformations of individual glycosidic linkages as a result of strong glycan-glycan or glycan-protein interactions (Figure 5). Results show no significant difference between lowest and highest cross-correlation frames in terms of the conformations assumed by the individual glycosidic linkages, that are all canonical, low energy ones. Thus, when enough density is available such as in positions N1098 and N1134, correlation with the cryo-EM map originates from the global glycan shape resulting from the combination of said linkage conformations, and the relative orientation of the glycan branches dictated by the geometry around the α-Man-(1→6)-β-Man bond, particularly the β-Man ω angle. The cryo-EM preferred value for ω is ∼−60°, corresponding to the most populated *gg* conformation for the free glycan core in solution (Figure 5C), which is therefore the value assumed by this angle in the pool of highest real-space correlation frames. The pool of lowest real-space correlation frames presents a population distribution among the three possible conformations (*gg*, *tg*, and *gt*), with an increased presence of the *tg* conformation that is virtually absent in the free glycan. Results for the other types of chemical modifications (Supplementary Figures 10, 11) are in line with those shown in Figure 5. When the glycans are less visible, the density does not carry information on the orientation of the branch connected to the α-Man-(1→6)-β-Man bond, and highest CC frames show a distribution of ω angles similar to that of the free glycan. Finally, the geometrical similarity of the highest and lowest cross-correlation (CC) frames was characterized by calculating the heavy-atom RMSD of the glycan core of each frame using as reference the global cross-correlation maximum (Supplementary Figure 12). The highest correlation frames lie in a narrow RMSD range over the global maximum, while the lowest correlation frames fall at larger (often > 10 Å) RMSD value, demonstrating that cross-correlation is a function of the glycan core geometry.

## Conclusion

Glycan mobility has several implications in many physiological processes. In the attempt to reconcile the apparently more ordered glycans on the S2 domain of SARS-CoV-2 spike protein provided by the cryo-EM density with their dynamics derived from MD simulations, we investigated the glycan poses that best would fit the density and assessed the effect of sugar chemical modifications on the model-map correlation. While those poses that best fit the map are as energetically viable as those that do not fit the map, according to the geometry of the core glycosidic bonds, we noted that the best fits are characterized by sugars showing very similar and canonical geometries around the α-Man-(1→6)-β-Man bond. This observation may suggest that the best model-map fits may recapitulate those poses for which the movements of the most external carbohydrates are more geometrically restricted, and it may explain why some of those sugars are visible in the cryo-EM map beyond the fact that the proteinaceous region of attachment is rigid.

In conclusion, integrating static and dynamic views remains challenging particularly in the case of glycoproteins for which experimentally derived densities are difficult to interpret and rationalize and computer modeling is limited by insufficient conformational sampling. This study, however, shows that experimental and computational tools combined can provide valuable insights on the conformational preferences of inherently flexible and complex glycoconjugates.

## Data Availability Statement

The original contributions presented in the study are included in the article/Supplementary Material, further inquiries can be directed to the corresponding authors.

## Author Contributions

NA and GJ-O conceived the study. AM-C, DC, OM, CB, AP, and JE-O helped throughout the biochemical tasks. SS produced and purified biological and prepared sample for cryo-EM and together with SC and NA collected cryo-EM data. SS, SC, and NA processed the images and interpreted cryo-EM maps. FP and GJ-O performed MD simulations, and with AA and JJ-B interpreted MD results. SS, SC, FP, NA, and GJ-O analyzed and contextualized all data and wrote the manuscript. All authors approved the final findings presented.

## Conflict of Interest

The authors declare that the research was conducted in the absence of any commercial or financial relationships that could be construed as a potential conflict of interest.

## Publisher’s Note

All claims expressed in this article are solely those of the authors and do not necessarily represent those of their affiliated organizations, or those of the publisher, the editors and the reviewers. Any product that may be evaluated in this article, or claim that may be made by its manufacturer, is not guaranteed or endorsed by the publisher.
