# Supplementary material for: Assessing the Mobility of Severe Acute Respiratory Syndrome Coronavirus-2 Spike Protein Glycans by Structural and Computational Methods
Source: Front Microbiol. 2022 Apr 15;13:870938. doi: 10.3389/fmicb.2022.870938 (PMC9053831; doi:10.3389/fmicb.2022.870938)
Supplement: Supplementary file 1 [file Data_Sheet_1.pdf]

SUPPLEMENTARY INFORMATION

Assessing the mobility of SARS-CoV-2 spike protein glycans by structural and computational methods

Soledad Stagnoli[1†], Francesca Peccati[2†], Sean R Connell[1,3,4], Ane Martinez-Castillo[1], Diego Charro[1], Oscar Millet[5], Chiara Bruzzone[5], Asis Palazon[3,6], Ana Ardá[3,7], Jesús Jiménez-Barbero[3,7], June Ereño-Orbea[3,7], Nicola GA Abrescia[1,3,8] *, Gonzalo Jiménez-Osés[2,3] *.

[1]Structure and Cell Biology of Viruses Lab, Center for Cooperative Research in Biosciences (CIC bioGUNE), Basque Research and Technology Alliance (BRTA), Bizkaia Technology Park, Derio, Spain.

[2]Computational Chemistry Lab, CIC bioGUNE, Basque Research and Technology Alliance (BRTA), Bizkaia Technology Park, Derio, Spain.

[3]IKERBASQUE, Basque Foundation for Science, Bilbao, Spain.

[4]Precision Medicine and Metabolism Laboratory, CIC bioGUNE, BRTA, Bizkaia Technology Park, Derio, Spain.

[5]Cancer Immunology and Immunotherapy Lab, CIC bioGUNE, BRTA, Bizkaia Technology Park, Derio, Spain.

[6]Chemical Glycobiology Laboratory, CIC bioGUNE, BRTA, Bizkaia Technology Park, Derio, Spain.

[7]Centro de Investigación Biomédica en Red de Enfermedades Hepáticas y Digestivas (CIBERehd), Instituto de Salud Carlos III, Madrid.

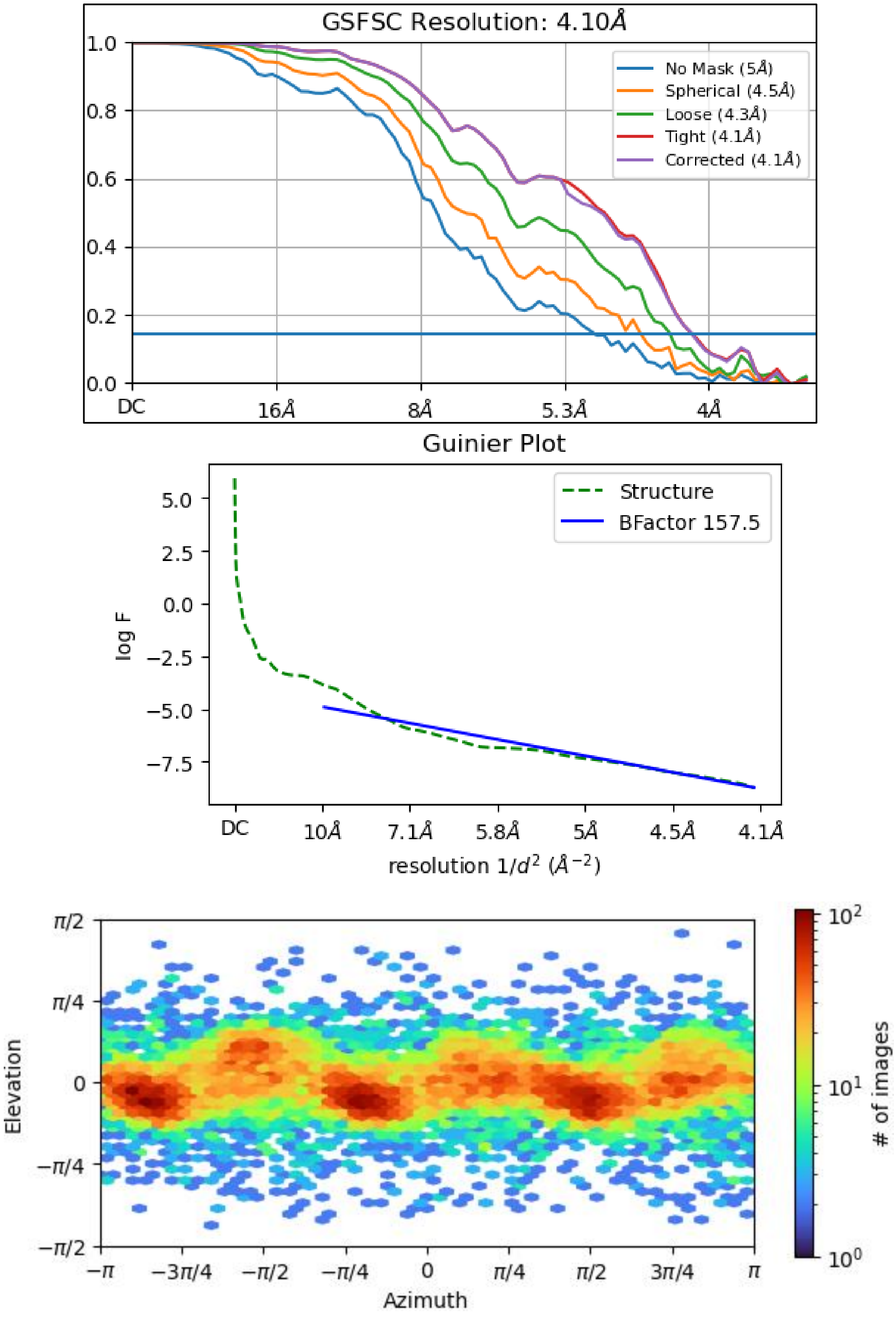

Supplementary Figure 1. Top, Fourier shell correlation (FSC) in CryoSPARC at the 0.143 criteria. The different colour lines indicate blue, no mask; orange, spherical mask; green, loose, soft mask; red, tight mask; magenta, corrected mask (for details see: https://discuss.cryosparc.com/t/tight-corrected-and-loose-gsfsc-curves/201/3). Centre, Guinier plot for Bfactor estimation. Bottom, orientation distribution map of the contributing particles.

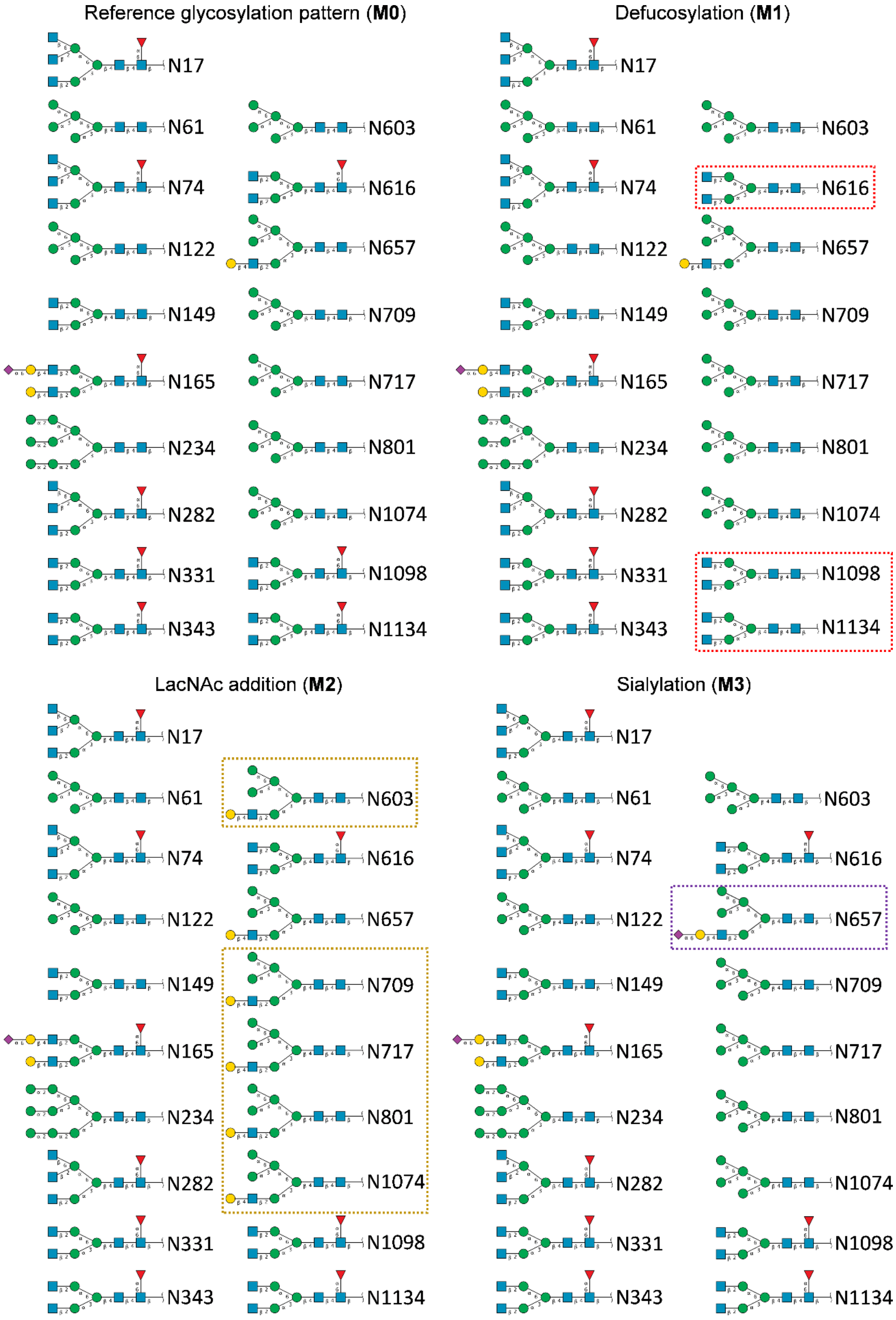

Supplementary Figure 2. Glycans modelled in the different glycosylated asparagine residues of spike protein S. Modified glycans with respect to the reference glycoprotein (M0) are highlighted.

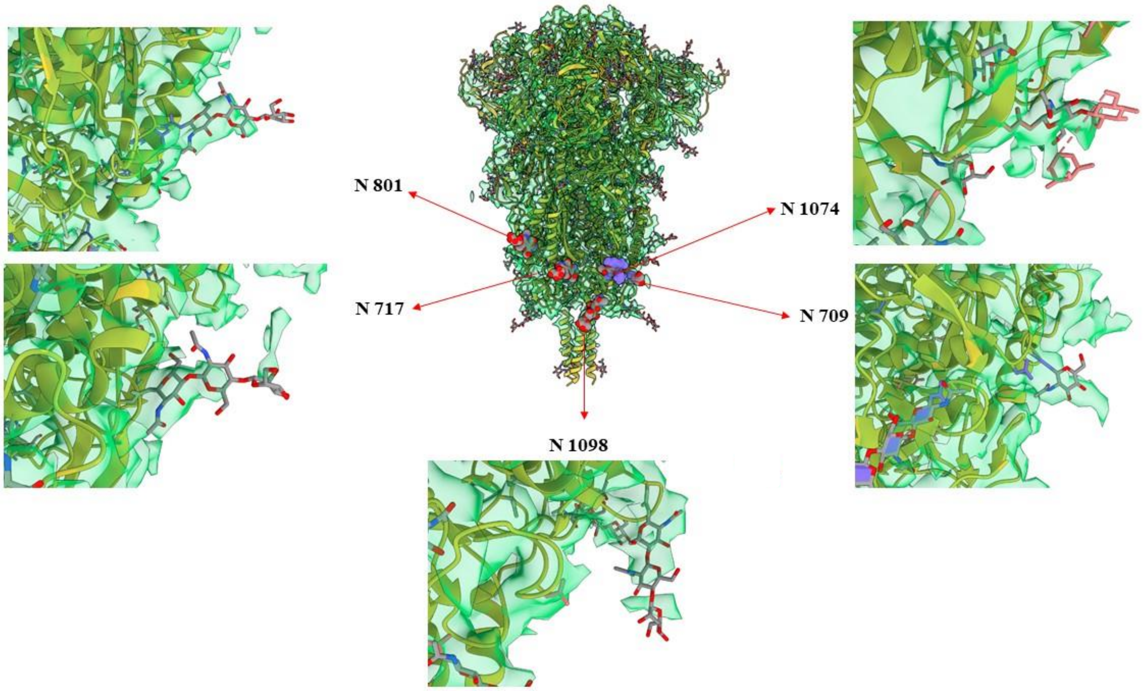

Supplementary Figure 3. As for Figure 1A but showing the visible density for other glycans (in sticks) present in the S2 domain of the spike protein.

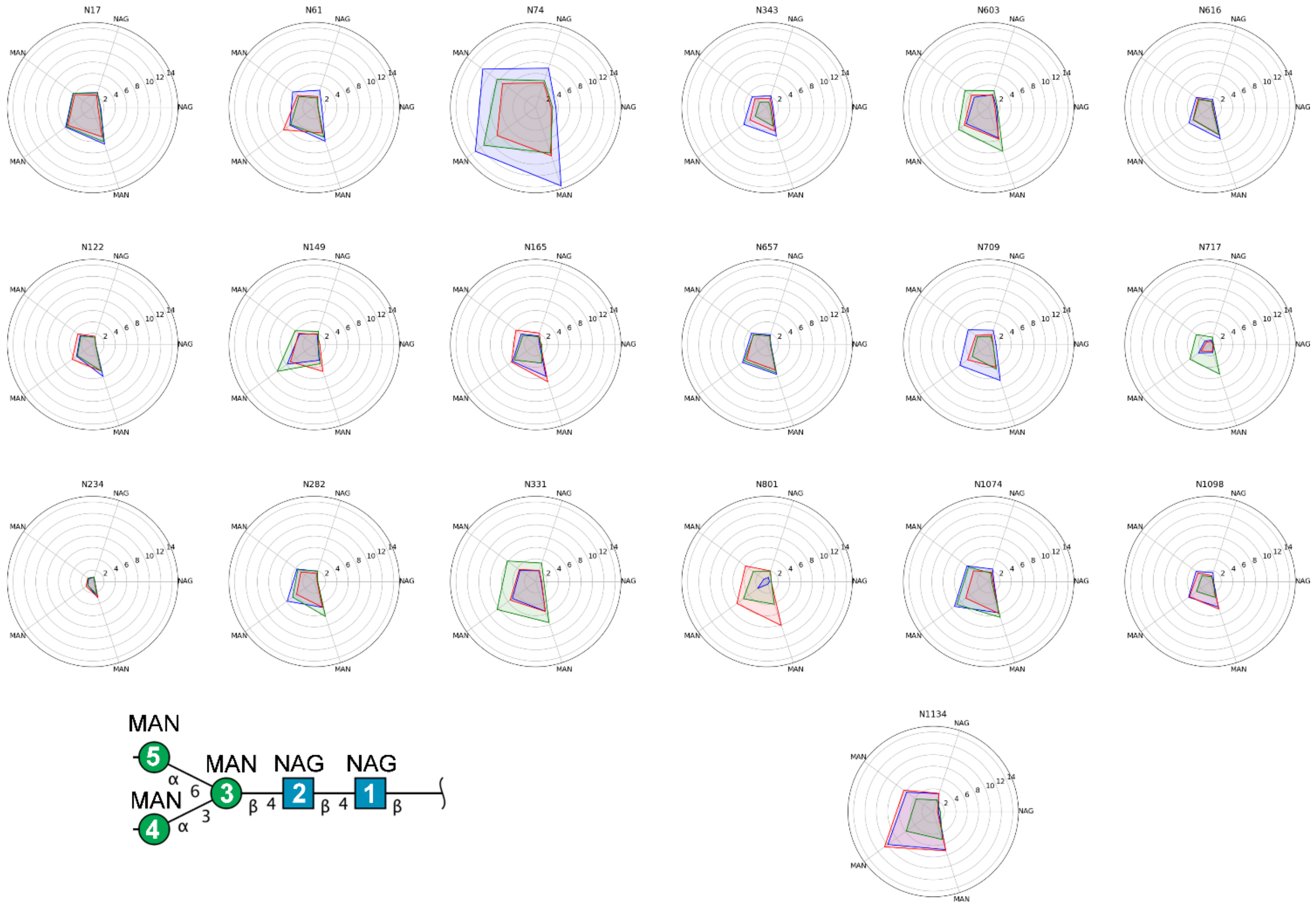

Supplementary Figure 4. Mobility of glycans from MD simulations for spike variant M0. For each glycosylation position, the mobility of core carbohydrates is computed as the atomic positional fluctuation. The first two N-acetylglucosamines (NAG) and three mannoses (MAN) – i.e. the glycan core – have been considered as they are shared among all glycans. Atomic fluctuations (RMSD in Å) are presented as radial plots. The mobility of the five carbohydrate units is represented anticlockwise starting from the first NAG. Chain A is represented in blue, chain B in red and chain C in green.

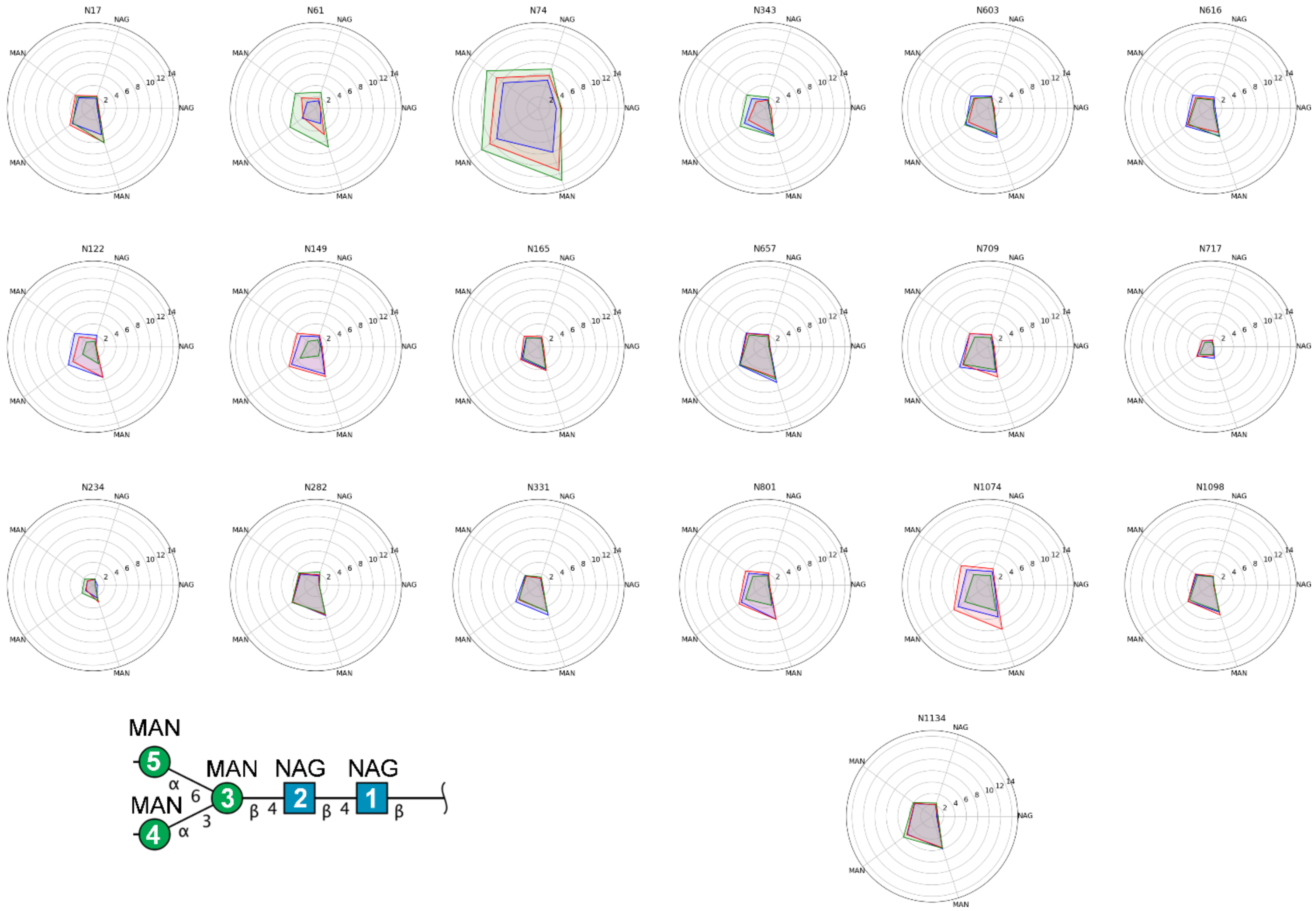

Supplementary Figure 5. Mobility of glycans from MD simulations for spike variant M1. For each glycosylation position, the mobility of core carbohydrates is computed as the atomic positional fluctuation. The first two N-acetylglucosamines (NAG) and three mannoses (MAN) – i.e. the glycan core – have been considered as they are shared among all glycans. Atomic fluctuations (RMSD in Å) are presented as radial plots. The mobility of the five carbohydrate units is represented anticlockwise starting from the first NAG. Chain A is represented in blue, chain B in red and chain C in green.

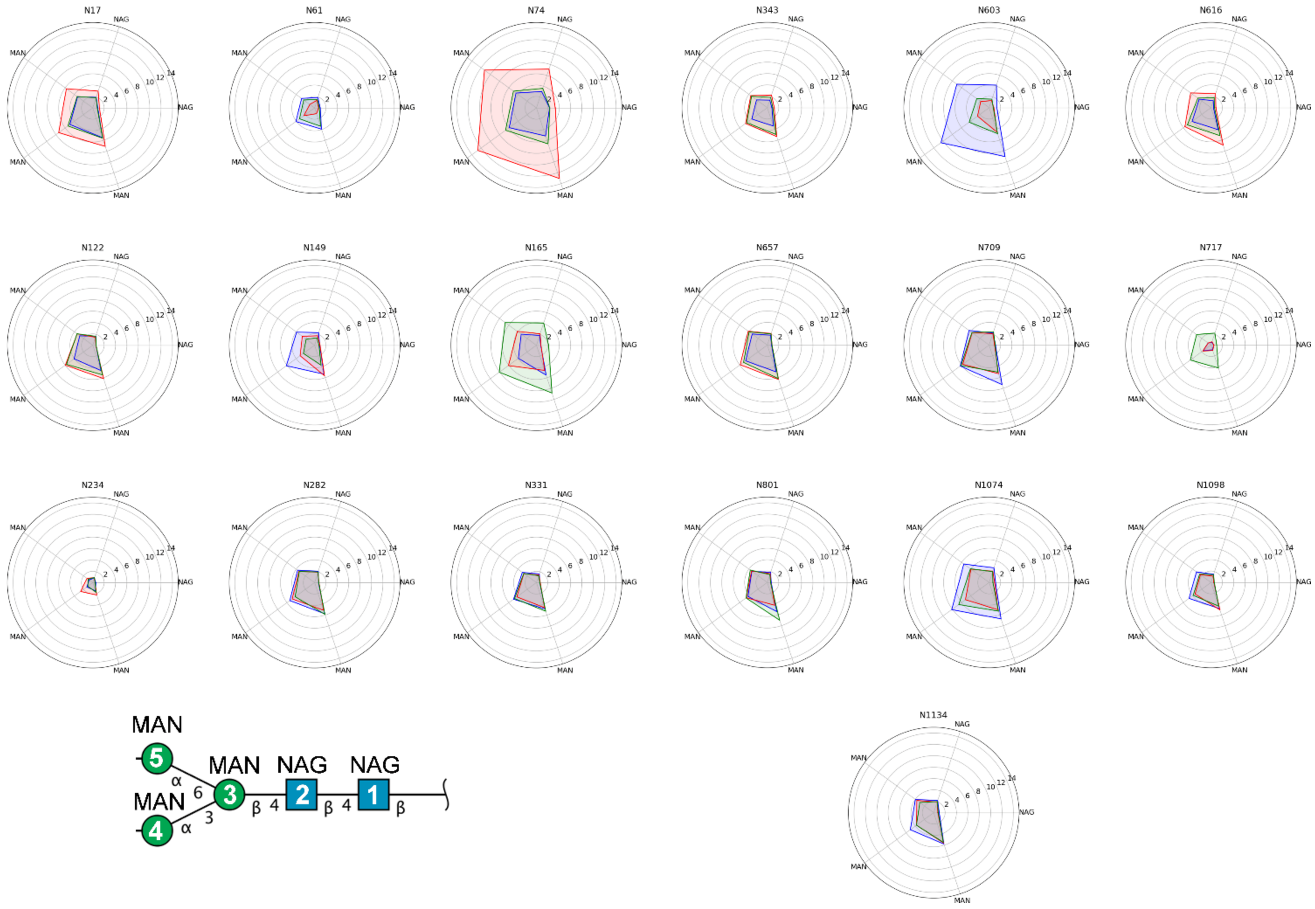

Supplementary Figure 6. Mobility of glycans from MD simulations for spike variant M2. For each glycosylation position, the mobility of core carbohydrates is computed as the atomic positional fluctuation. The first two N-acetylglucosamines (NAG) and three mannoses (MAN) – i.e. the glycan core – have been considered as they are shared among all glycans. Atomic fluctuations (RMSD in Å) are presented as radial plots. The mobility of the five carbohydrate units is represented anticlockwise starting from the first NAG. Chain A is represented in blue, chain B in red and chain C in green.

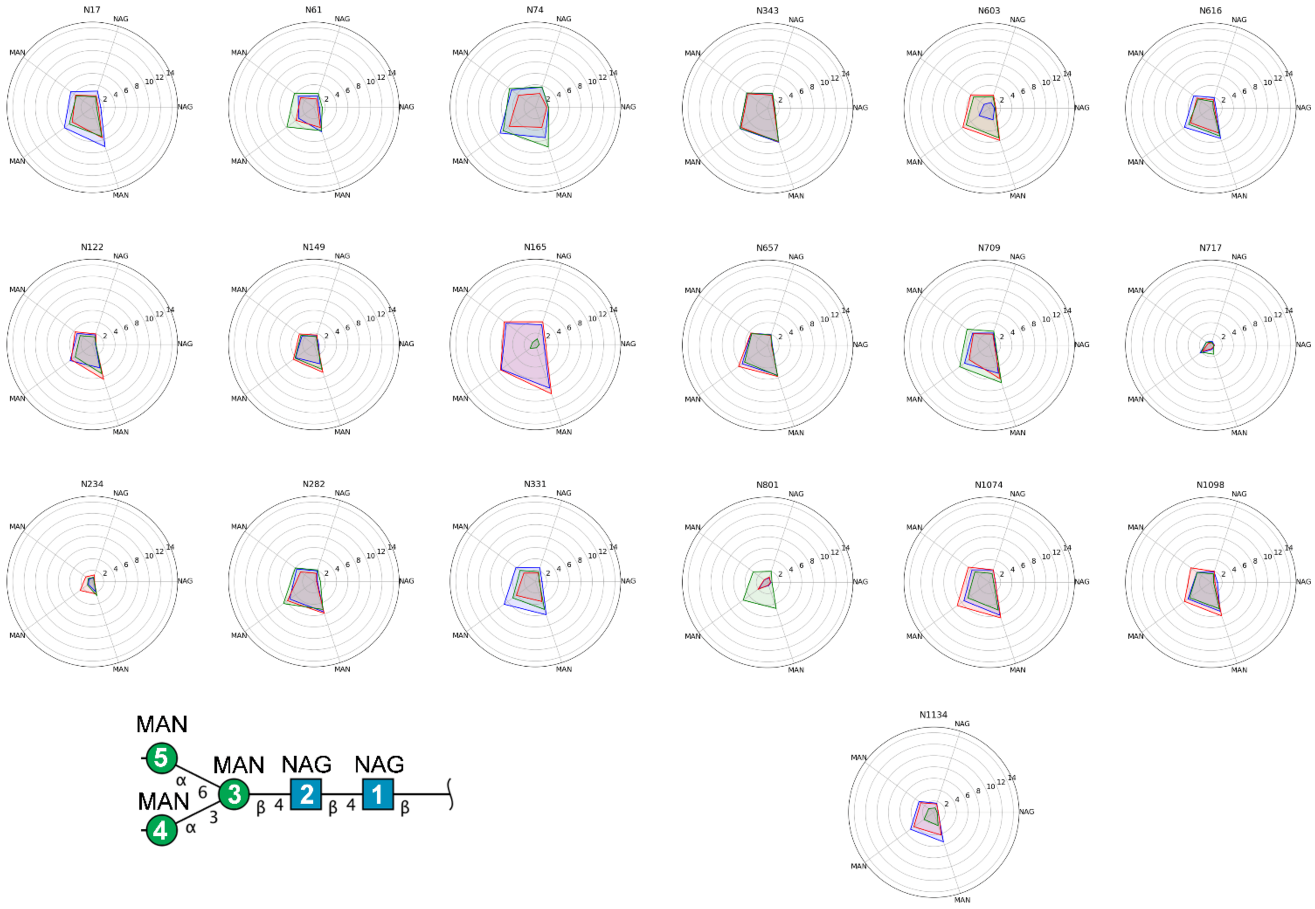

Supplementary Figure 7. Mobility of glycans from MD simulations for spike variant M3. For each glycosylation position, the mobility of core carbohydrates is computed as the atomic positional fluctuation. The first two N-acetylglucosamines (NAG) and three mannoses (MAN) – i.e. the glycan core – have been considered as they are shared among all glycans. Atomic fluctuations (RMSD in Å) are presented as radial plots. The mobility of the five carbohydrate units is represented anticlockwise starting from the first NAG. Chain A is represented in blue, chain B in red and chain C in green.

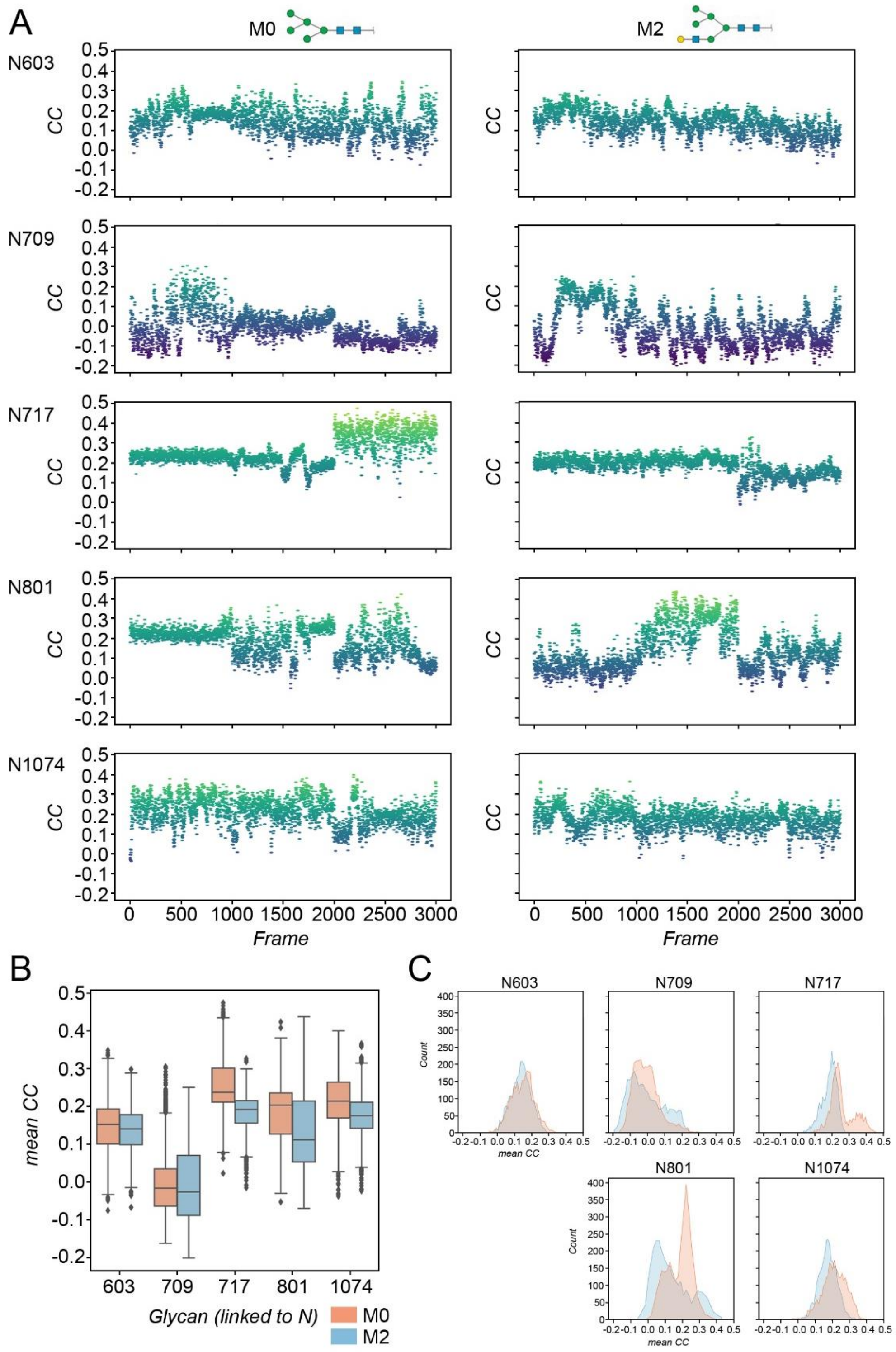

Supplementary Figure 8. (A) Plots of the real-space correlation (CC) of the glycans linked to N603, N709, N717, N801 and N1074 across all the 3,000 frames (each monomer is considered independent) for the model M0 (left) and M2 (right, addition of terminal LacNAc). **(B)** Histograms of the above cross correlation values. **(C)** Box plots showing the distribution of the above cross correlation.

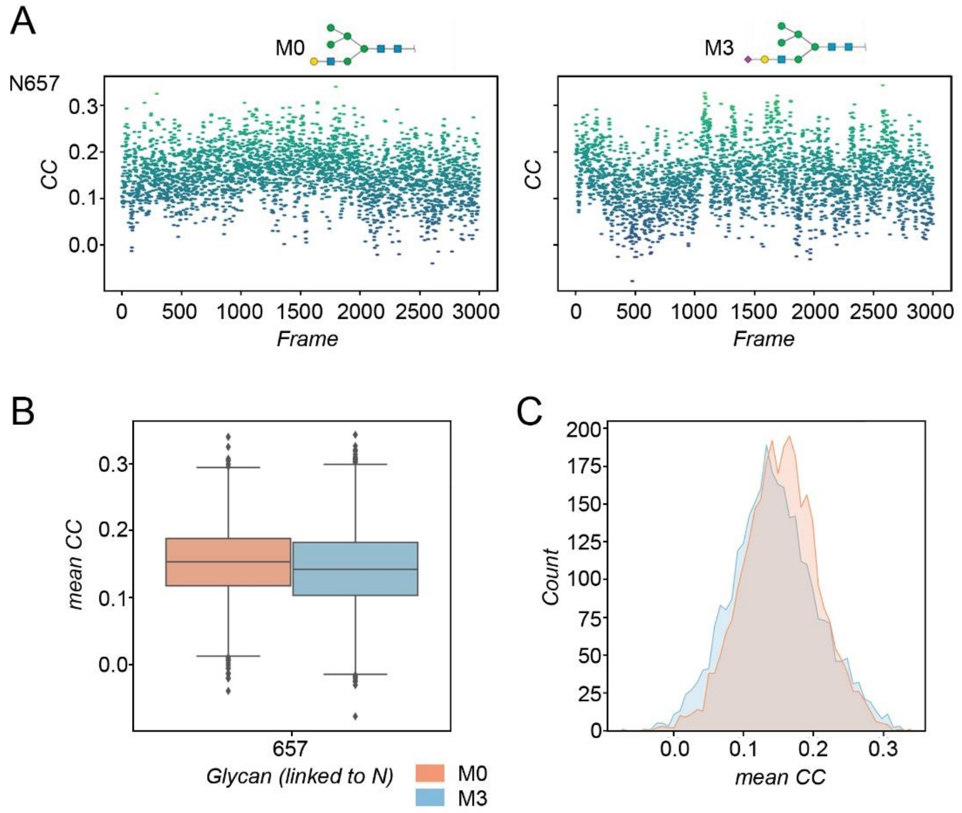

Supplementary Figure 9. (A) Plots of the real-space correlation (CC) of the glycans linked to N657, across all the 3,000 frames (each monomer is considered independent) for the model M0 (left) and M3 (right, sialylation). **(B)** Histograms of the above cross correlation values. **(C)** Box plots showing the distribution of the above cross correlation.

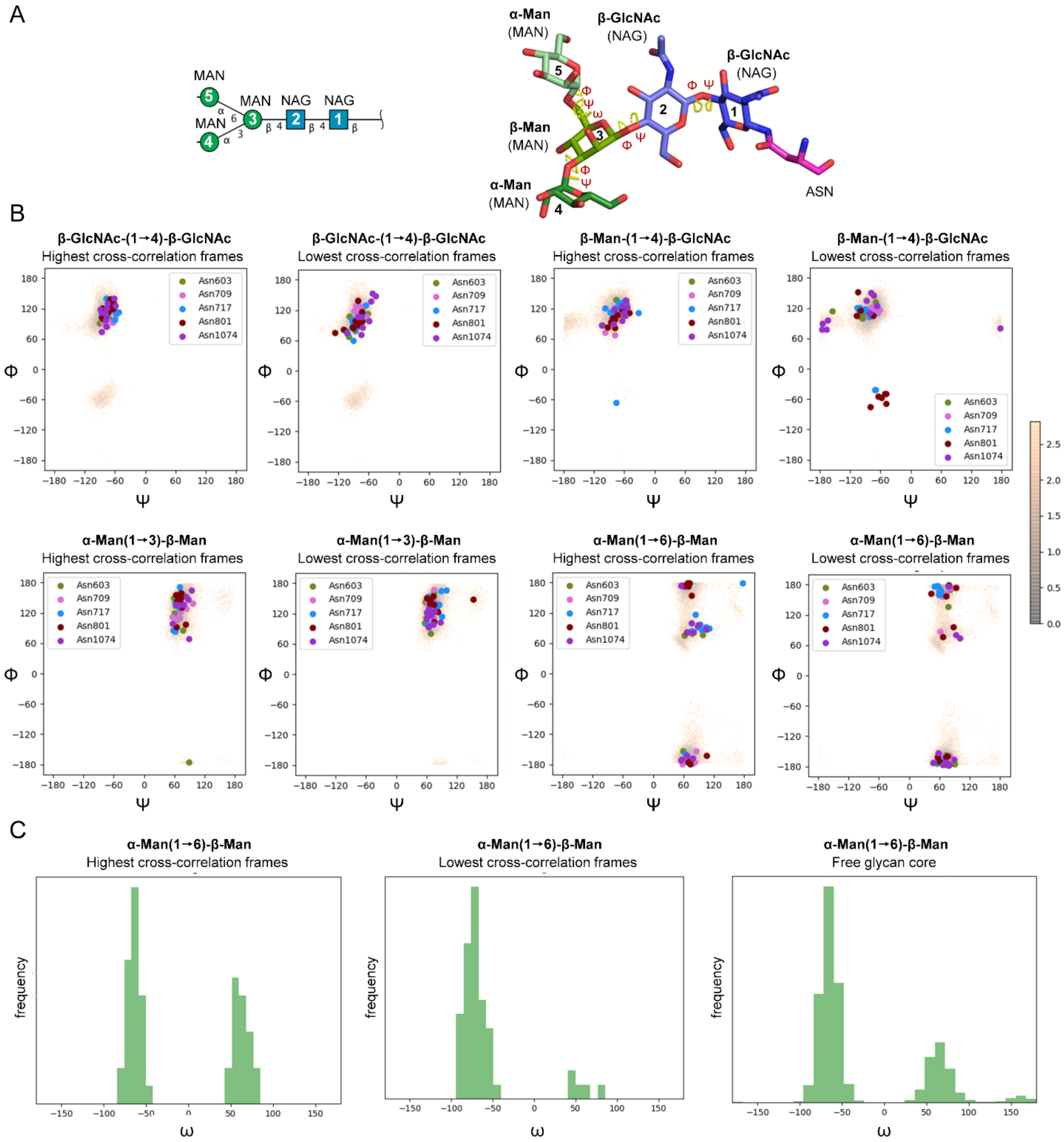

Supplementary Figure 10. (A) Symbol Nomenclature for Glycans (SNFG) and three-dimensional representations of the glycan core analyzed by MD simulations. **(B)** Φ/Ψ plots for the glycosidic bonds in the glycan core of selected glycosylation positions (N603, N709, N717, N801, N1074) corresponding to MD snapshots that scored the highest (n=10) and the lowest (n=10) real-space cross-correlation (CC) with cryo-EM density (colored circles), and the free glycan core in solution (density map color-coded according to conformational energy in kcal mol^{-1} derived from population analysis though a Boltzmann distribution at 25 °C). (**C**) Histograms for the ω dihedral angle in the branching mannose of the glycan core of selected glycosylation positions (N603, N709, N717, N801, N1074) corresponding to MD snapshots that scored the highest (n=10) and the lowest (n=10) real-space cross-correlation (CC) with cryo-EM density, and the free glycan core in solution.

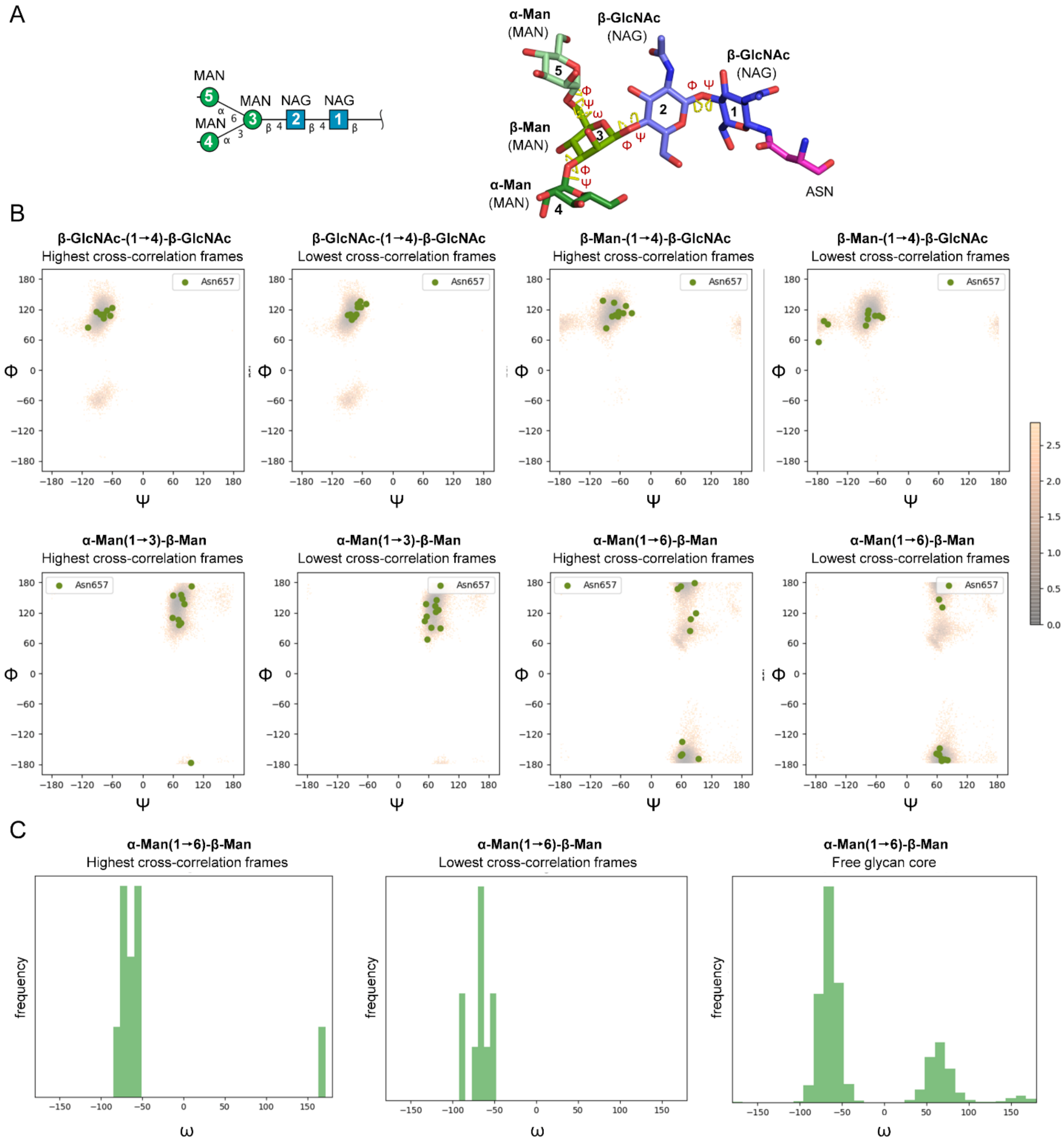

Supplementary Figure 11. (A) Symbol Nomenclature for Glycans (SNFG) and three-dimensional representations of the glycan core analyzed by MD simulations. **(B)** Φ/Ψ plots for the glycosidic bonds in the glycan core of selected glycosylation positions (N657) corresponding to MD snapshots that scored the highest (n=10) and the lowest (n=10) real-space cross-correlation (CC) with cryo-EM density (colored circles), and the free glycan core in solution (density map color-coded according to conformational energy in kcal mol^{-1} derived from population analysis though a Boltzmann distribution at 25 °C). (**C**) Histograms for the ω dihedral angle in the branching mannose of the glycan core of selected glycosylation positions (N657) corresponding to MD snapshots that scored the highest (n=10) and the lowest (n=10) real-space cross-correlation (CC) with cryo-EM density, and the free glycan core in solution.

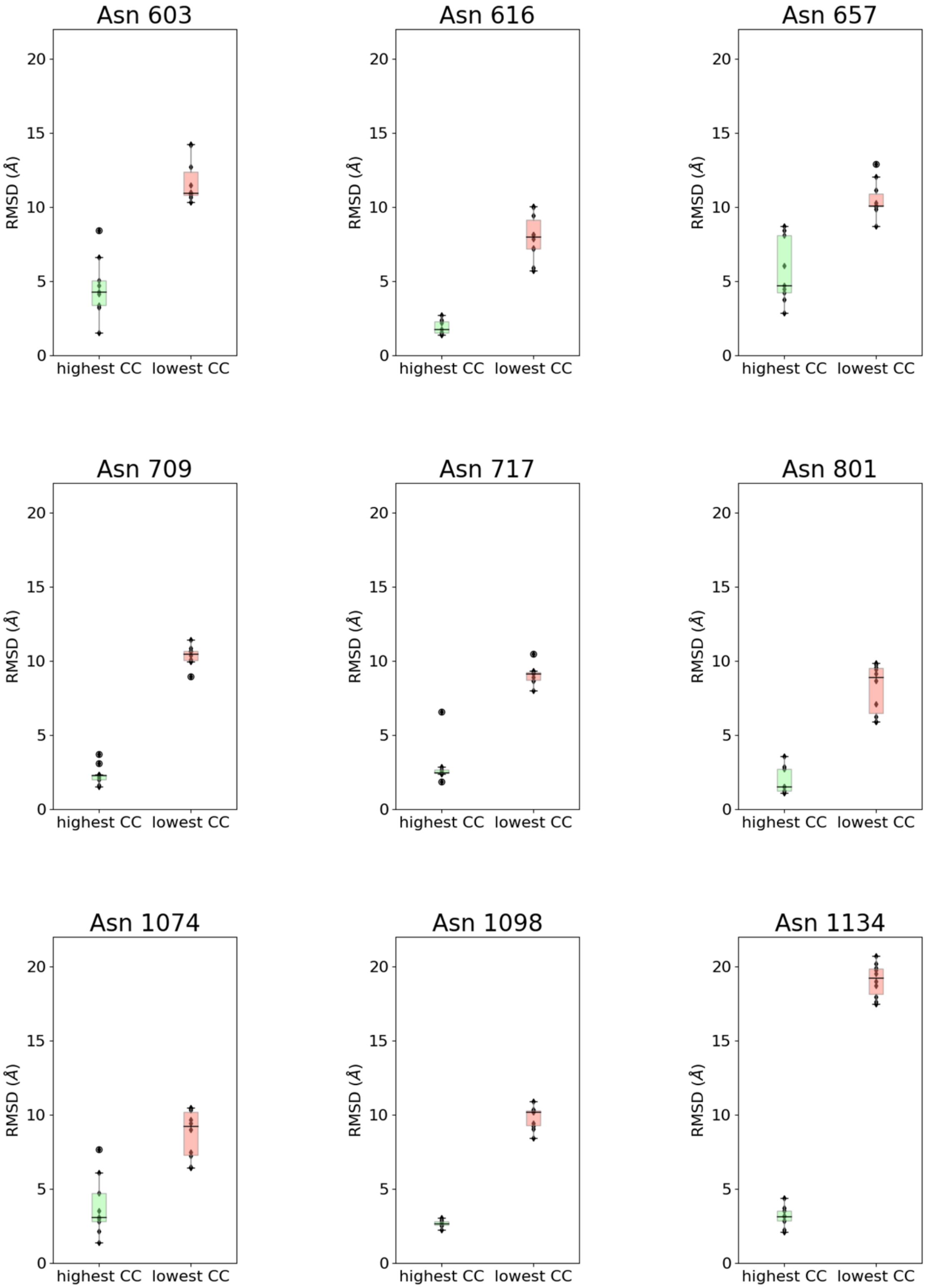

Supplementary Figure 12. Box plots of the root-mean-square deviations (RMSD) (Å) calculated for the glycan core of all selected glycosylation positions corresponding to MD snapshots that scored the highest (n=10, in green) and the lowest (n=10, in red) real-space cross-correlation (CC) with cryo-EM density, calculated using as reference the highest cross correlation frame; the null RMSD value of the reference frame is thus omitted.

Supplementary Table 1. Cryo-EM data collection, refinement, and validation statistics for ecto-S

**Data collection**	*dataset A*	*dataset B*
Nominal magnification	105,000	
Voltage (kV)	300	
Electron exposure ($e^{-}/Å^2$)	49.6	50
Defocus range (μm)	-0.5 to -3.5	
Sampling interval (Å/pixel)	0.83	
Frames	40	50
N° movies	12,478	9,254
**Cryo-EM processing**	*RELION /cryoSPARC*	
Contributing particles	22,854	
Box Size (pixel)	192	
Pixel size (Å)	1.66	
Symmetry	C3	
Map resolution (Å)<br>FSC threshold	4.1<br>0.143	
Map sharpening *B* factor ($Å^2$)	-157.5	
EMDB code	EMD-14621	
**Structure refinement**		
PDB ID used	6XR8	
CC, (mask)	0.71	
CC, (box)	0.80	
Resolution Estimates (Å)		
d99	4.4	
d FSC model (0.143)	4.0	

Supplementary Table 2. Size of the simulation box (fixed in the NVT ensemble) and maximum distance between all pairs of C1 atoms of terminal glycans along the three Cartesian coordinates (representing the size of the glycoprotein) throughout the MD simulation of spike protein variant M0. All dimensions are given in Å.

	Size along X	Size along Y	Size along Z
Simulation box	*182.577*	*175.607*	*210.987*
**5 ns**	158.276	145.991	161.016
**10 ns**	150.784	147.108	162.555
**15 ns**	153.471	150.281	163.179
**20 ns**	143.216	148.394	162.005
**25 ns**	153.827	148.402	156.521
**30 ns**	155.240	143.420	159.431
**35 ns**	153.334	149.144	161.678
**40 ns**	145.259	142.407	158.617
**45 ns**	151.166	135.698	161.479
**50 ns**	145.723	133.624	162.105
**55 ns**	138.877	128.307	158.732
**60 ns**	142.978	129.863	162.041
**65 ns**	140.725	133.791	158.634
**70 ns**	141.876	133.863	156.706
**75 ns**	148.738	139.122	156.419
**80 ns**	147.520	137.425	159.038
**85 ns**	150.883	138.712	161.232
**90 ns**	151.588	132.927	159.897
**95 ns**	150.174	134.230	158.568
**100 ns**	152.596	135.306	159.607

Supplementary Table 3. Size of the simulation box (fixed in the NVT ensemble) and maximum distance between all pairs of C1 atoms of terminal glycans along the three Cartesian coordinates (representing the size of the glycoprotein) throughout the MD simulation of spike protein variant M1. All dimensions are given in Å.

	Size along X	Size along Y	Size along Z
Simulation box	*186.783*	*179.53*	*208.103*
**5 ns**	155.757	150.495	159.228
**10 ns**	160.429	145.507	156.134
**15 ns**	157.846	143.075	162.515
**20 ns**	148.840	139.586	156.990
**25 ns**	143.394	146.844	156.303
**30 ns**	144.886	143.929	155.982
**35 ns**	143.326	145.233	156.566
**40 ns**	150.549	139.412	157.746
**45 ns**	147.277	141.453	156.195
**50 ns**	149.983	147.320	155.890
**55 ns**	135.437	143.794	158.492
**60 ns**	141.268	132.519	154.801

**65 ns**	139.046	131.081	163.116
**70 ns**	142.669	134.490	166.250
**75 ns**	148.397	137.196	162.926
**80 ns**	148.074	133.822	157.305
**85 ns**	127.710	139.974	156.308
**90 ns**	137.613	133.687	160.300
**95 ns**	142.581	148.548	156.931
**100 ns**	141.707	148.105	156.708

Supplementary Table 4. Size of the simulation box (fixed in the NVT ensemble) and maximum distance between all pairs of C1 atoms of terminal glycans along the three Cartesian coordinates (representing the size of the glycoprotein) throughout the MD simulation of spike protein variant M2. All dimensions are given in Å.

	**Size along X**	**Size along Y**	**Size along Z**
Simulation box	*186.814*	*179.560*	*208.138*
**5 ns**	162.858	149.013	162.684
**10 ns**	155.942	150.238	157.179
**15 ns**	154.110	150.729	159.374
**20 ns**	154.127	143.615	155.505
**25 ns**	158.194	148.384	161.013
**30 ns**	149.999	146.984	165.186
**35 ns**	151.706	148.320	160.265
**40 ns**	150.787	143.475	162.470
**45 ns**	151.190	144.895	163.385
**50 ns**	148.335	147.832	157.119
**55 ns**	151.250	148.241	162.609
**60 ns**	149.521	148.663	158.223
**65 ns**	151.906	144.033	155.874
**70 ns**	146.346	139.666	161.412
**75 ns**	153.106	144.845	158.264
**80 ns**	150.139	144.479	158.467
**85 ns**	155.315	148.270	160.260
**90 ns**	151.285	145.019	159.770
**95 ns**	151.896	148.738	160.966
**100 ns**	152.266	141.992	158.826

Supplementary Table 5. Size of the simulation box (fixed in the NVT ensemble) and maximum distance between all pairs of C1 atoms of terminal glycans along the three Cartesian coordinates (representing the size of the glycoprotein) throughout the MD simulation of spike protein variant M3. All dimensions are given in Å.

	**Size along X**	**Size along Y**	**Size along Z**
Simulation box	*187.566*	*176.25*	*203.388*
**5 ns**	158.353	154.281	161.476
**10 ns**	151.076	149.189	161.679
**15 ns**	145.350	144.085	159.893

**20 ns**	149.577	146.700	165.428
**25 ns**	140.731	138.180	167.479
**30 ns**	135.866	137.681	166.007
**35 ns**	143.416	141.792	160.288
**40 ns**	137.198	138.214	159.920
**45 ns**	135.661	140.241	160.106
**50 ns**	152.071	141.101	161.185
**55 ns**	148.241	140.955	163.437
**60 ns**	141.138	141.803	163.454
**65 ns**	142.940	141.923	160.208
**70 ns**	142.306	142.630	159.823
**75 ns**	143.802	142.475	165.358
**80 ns**	143.550	141.231	163.190
**85 ns**	143.345	137.630	162.955
**90 ns**	146.497	142.471	162.326
**95 ns**	145.315	138.904	159.817
**100 ns**	143.579	144.062	160.615

Supplementary Table 6. Root-mean-square deviation (RMSD) (Å) between initial geometry and geometry after MD equilibration of the of the four spike protein variants, considering i) all protein atoms, ii) all glycan atoms and iii) all glycoprotein atoms.

Variant	Protein	Glycans	Protein + Glycans
M0	1.417	8.039	3.624
M1	0.944	6.858	2.968
M2	0.918	7.191	3.174
M3	0.994	7.033	3.067

Supplementary Table 7. Glycan shielding (GS) in spike protein variants with respect to the non-glycosylated protein. GS is calculated as the percentage of the solvent accessible surface area (SASA) covered by glycans throughout MD simulations of each variant.

Variant	SASA reduction (%)
M0	–12.8 ± 0.4
M1	–12.9 ± 0.3
M2	–13.8 ± 0.3
M3	–14.0 ± 0.4
